# Brain Metastasis of Follicular Thyroid Carcinoma in Pregnancy: A Case Report and Literature Review

**DOI:** 10.7759/cureus.9337

**Published:** 2020-07-22

**Authors:** Erlan P Lopes Rufino, Eduarda S Ribeiro da Costa Gomes, Lucas M Silva Loureiro, Pierre Oliveira Eugenio, Francisco Vaz-Guimaraes

**Affiliations:** 1 Neurological Surgery, Real Hospital Portugues, Recife, BRA; 2 Faculty of Medicine, Catholic University of Pernambuco, Recife, BRA; 3 Faculty of Medical Sciences of the University of Pernambuco, Hospital Universitário Oswaldo Cruz, Recife, BRA; 4 Neurological Surgery, Hospital Da Restauracao, Recife, BRA

**Keywords:** brain metastasis, surgical resection, follicular thyroid carcinoma, pregnancy

## Abstract

Brain metastases (BMs) related to cancer are quite common and represent the most common brain cancer. We present a rare case of a 32-year-old female, 36 weeks pregnant, admitted to the emergency with complaints of severe headache, vomiting, and left hemiparesis associated with drowsiness. Cranial tomography showed an image suggestive of an expansive lesion in the right front-temporo-insular region with an important mass effect. The result of biopsy with immunohistochemistry was compatible with metastasis of follicular thyroid carcinoma (FTC). The knowledge of neurological characteristics in the clinical analysis of patients with thyroid carcinoma must be highly valued, both in the correct interpretation of the signs and in the early investigation through skull imaging exams.

## Introduction

Brain metastases (BMs) are the most common neurological complication related to cancer and represent the most frequent brain cancer [1]. BMs occur in 15%-30% of cancer patients and cause significant morbidity and mortality. Its incidence has been increasing, both due to the better diagnosis of small lesions detected on MRI and better approach to systemic extracerebral disease [2]. Although CT is the initial exam in the treatment of brain diseases, MRI is still the best imaging method for the diagnosis of these metastases [3]. The most common tumors that cause BM are lung carcinomas (20%), melanoma (6.9%), kidney cancer (6.5%), breast (5.1%) and colorectal (1.8%), with differentiated thyroid cancer accounting for only 0.9% of all cases [4-5]. These metastases may be the first manifestation of cancer and the symptoms appear to be related to an expanding tumor mass and associated edema. The most frequent presentations of this complication include focal deficits, seizures, cognitive changes, and headache [4]. As for the management of BM, the three main treatment options are surgery, conventional radiotherapy and radiosurgery, used alone or in combination [2]. Follicular thyroid carcinoma (FTC) is the second most common thyroid cancer subtype, after papillary thyroid carcinoma (PTC), and is responsible for approximately 10% of all thyroid cancers [6]. BMs are a rare complication of FTC, but with significant mortality, making it clear the importance of investigating this site as a neoplastic origin in the clinical follow-up of the patient, as this scenario can be even more challenging to be managed during pregnancy [7].

This article presents a rare case of a pregnant patient who developed brain metastasis due to FTC, undergoing two distinct and concurrent surgical procedures (cesarean section and tumor excision) with a multidisciplinary team.

## Case presentation

**Figure 1:**
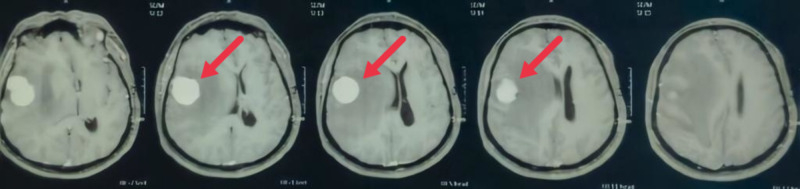
Skull CT with right front-temporo-insular lesion.

A 32-year-old female, 36 weeks pregnant, was admitted to the ER with complaints of severe headache, vomiting, and left hemiparesis associated with drowsiness. The patient was submitted to a cranial tomography that showed an image suggestive of an expansive lesion in the right front-temporo-insular region with an important mass effect (Figure 1).

Venous corticosteroid (dexamethasone 4 mg, four times a day) was administered with significant decrease in pain and nausea and submitted to imaging exams for etiological investigation (cranial resonance, tomography of upper, lower abdomen and chest). CT scans showed no suspected primary site lesions. After two days of in-hospital investigation, the patient developed a seizure, followed by a lower level of consciousness, requiring an orotracheal intubation. Emergency skull CT scan showed worsening of the edema with increased tumor mass effect.

**Figure 2:**
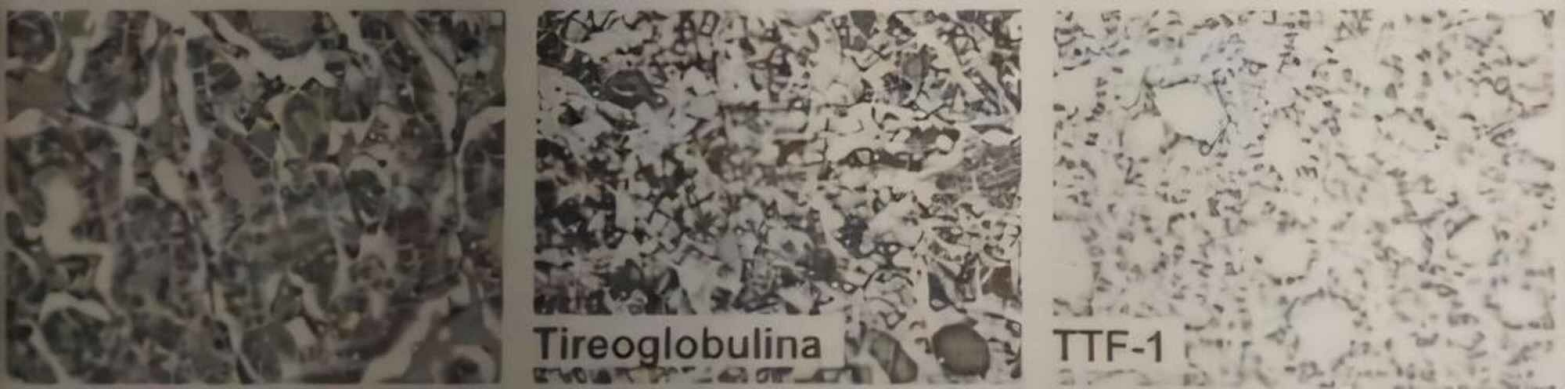
Histological sections of supratentorial lesion reveal fragments of neoplasia with a follicular architectural pattern, presence of cuboidal epithelial cells, and expression for PAX-8, thyroglobulin, and TTF-1. PAX-8, paired box gene 8; TTF-1, thyroid transcription factor 1

Due to the neurological surgical urgency, it was decided to call an obstetrics team to perform an emergency cesarean section followed by decompressive craniectomy associated with tumor excision. The patient was submitted to general anesthesia with the necessary care regarding anesthetic medications; cesarean section was performed without complications and the child was born well, active and reactive. During surgery for tumor excision, it was noticed that it was quite friable and with excessive bleeding facility. It was necessary for two red blood cell transfusions during surgery. Complete exeresis was performed together with decompressive craniectomy. The patient evolved with an improvement in the level of consciousness in the ICU after seven days, being discharged to the ward. She was discharged after two days, conscious and oriented, but still with an incomplete strength deficit in the left dimidium. The result of biopsy with immunohistochemistry was compatible with metastasis of FTC, with expression of paired box gene 8 (PAX-8), thyroglobulin, and thyroid transcription factor 1 (TTF-1) in fragments of supratentorial lesion (Figure 2). 

The patient underwent clinical follow-up with an oncology team. Radiotherapy was requested after the MRI showed a nodular area of enhancement by the contrasting substance in the left frontal subcortical region, with a maximum diameter of 0.5 cm, promoting perilesional edema, which in the clinical context represents a secondary lesion.

## Discussion

Differentiated thyroid carcinoma (DTC) arises from follicular cells and represents more than 90% of diagnosed cases of thyroid cancer; in this cancer group, papillary carcinoma is the most common (90%), followed by follicular carcinoma (10%) [8]. Both are associated with a favorable prognosis, with a 10-year survival rate of 80%-90%. However, although they are very similar, they behave differently. The follicular, subtype presented by the patient in the case, tends to be more aggressive, has higher mortality rates and is, more often, diagnosed in more advanced stages of the disease when compared with papillary carcinoma [9]. Still under this comparison, FTC is even more vascularized and, therefore, releases metastases more frequently, in a rate of 15%-27% of patients and the most affected sites are the bones and lungs [6]. In contrast, metastases can also occur in other soft tissues and in unusual locations; there are, for example, reports of FTC metastases affecting the skin, kidneys, pancreas, adrenal gland and brain, as shown in case [10]. Metastatic thyroid carcinomas are uncommon, occurring only in 0.9% of cases of BMs [5,11]. Most of these BMs are diagnosed during the follow-up of the primary tumor, with a mean interval between the diagnosis and the identification of the BM of 2.9 years [12]. In the case in question, however, the clinical manifestations resulting from BM were the first findings. It is known that the presence of these distant metastases is related to a worse prognosis for DTC, with a decrease of 50% in the survival rate at five years of follow-up in most series [13-15]. BM in thyroid cancer patients has been associated with men, advanced age, large primary tumors, and evidence of extra thyroid invasion with the appearance of distant metastases, which makes the reported case even more rare because it is a pregnant, young woman, with no evidence of large primary tumor or other distant metastasis site [12]. The most frequent location of BM is in the cerebral hemispheres, as occurred in the patient in the report, although they may also appear in the pituitary and cerebellum [9].

The treatment of choice is surgical resection of metastasis followed by radiotherapy [9]. In this case, due to the circumstances of a neurological surgical emergency, it was decided to perform an emergency cesarean section concomitant with a decompressive craniectomy associated with excision of the tumor. The conduct was successful and the patient evolved with an improvement in the symptoms initially presented, having clinical follow-up by the oncology and radiotherapy teams of the service. The prognosis of patients affected by BM secondary to DTC is generally poor and there is a consensus in the literature that metastasis is associated with greater chances of recurrence [16]. The clinical picture of DTC with brain metastasis is not well established, due to its low prevalence. Different reports in the literature in the last 10 years show that the symptoms of BM derived from DTC have different presentations, such as headache, nausea, motor and sensory deficits, diplopia, visual loss, ataxia, seizures, and others. This is clear from the table in question (Table 1).

**Table 1:** Comparative analysis between the clinical and therapeutic characteristics of DTCs with brain metastasis. A literature review was carried out in the PubMED database between 2010 and 2020, with the following keywords: brain metastasis and thyroid carcinoma. DTC, differentiated thyroid carcinoma; FTC, follicular thyroid carcinoma; PTC, papillary thyroid carcinoma

		Han et al. 2016 [12]		Taywade et al. 2016 [17]		Mori et al. 2016 [18]		Tanaka et al. 2013 [19]		Chhiber et al. 2011 [20]	
Gender		F		F		F		F		F	
Age		76 years		82 years		76 years		63 years		65 years	
Symptoms		Memory disturbances that had begun three months prior		The patient did not have symptoms related to pituitary involvement		Brain metastases were asymptomatic		Manifesting as lateral gazing nystagmus and slurred speech		The patient presented with sudden onset headache and bi-lateral loss of vision	
Metastasis site		Lobulated lesion in the right frontal lobe		Sella turcica in addition to multiple skeletal and lung metastases		Spinal intramedullary metastasis and multiple small brain metastases		Cerebellopontine angle		Sellar metastasis	
Outcome		Thyroidectomy and radiotherapy were recommended but the patient refused all treatment		Was decided to start the patient on high-dose radioiodine treatment		Both the spinal and brain metastases were successfully treated by frameless stereotactic radiotherapy and stereotactic radiosurgery		Left retrosigmoid craniotomy was performed. The patient then underwent adjuvant gamma knife radiosurgery		The patient underwent early trans-sphenoidal decompression	
Primary tumor		PTC		FTC		PTC		PTC		FTC	

In the case discussed, with the presence of a lesion with characteristics of an important mass effect, the patient presented complaints of nausea and headache. Such symptoms constituted the first presentation of the FTC, undiagnosed until histopathological investigation after the surgical procedure. The most common clinical presentation of FTC, in turn, is a single, painless thyroid nodule. Patients with largely invasive disease often complain of a palpable mass in the neck; while patients with minimally invasive disease are usually diagnosed with a palpable neck mass or diagnosed incidentally [6]. Despite this, as previously mentioned, none of these symptoms was reported by the patient in question, whose clinical presentation was at first restricted to intracranial involvement. The case presented is singular and rare in some aspects: metastasis of FTC (less common histological subtype), first symptoms were neurological (uncommon presentation), diagnosis after histopathological examination of the lesion (unusual diagnosis), and concomitance with pregnancy.

## Conclusions

The knowledge of neurological characteristics in the clinical analysis of patients with thyroid carcinoma must be highly valued, both in the correct interpretation of the signs and in the early investigation through skull imaging exams. Further, it is essential to perform early surgery in order to avoid the inherent mass effects of the expanding tumor. Therefore, FTC, even though it has a low incidence of brain metastasis, should be remembered in patients investigating a primary tumor site in order to have a better therapeutic plan and clinical follow-up.
